# Elastoplastic Modeling of Kevlar^®^ Composite Laminates: A Cyclic Loading Approach for In-Plane Characterization

**DOI:** 10.3390/polym17162235

**Published:** 2025-08-17

**Authors:** Rene Alejandro Canceco de la Cruz, Luis Adrián Zúñiga Avilés, Gabriel Plascencia Barrera, Alberto Díaz Díaz, José Martin Herrera Ramírez

**Affiliations:** 1 Centro de Investigación en Materiales Avanzados, S.C. (CIMAV), Av. Miguel de Cervantes #120, Complejo Industrial Chihuahua, Chihuahua 31136, Mexico; rene.canceco@cimav.edu.mx (R.A.C.d.l.C.); gabriel.plascencia@cimav.edu.mx (G.P.B.); 2 Facultad de Medicina, Universidad Autónoma del Estado de México, Toluca 50180, Mexico; adriandgim@gmail.com

**Keywords:** Kevlar^®^, reinforced polymer composite, laminate, nonlinear behavior, mechanical test, equivalent plastic strain

## Abstract

This study investigates the elastoplastic behavior of phenol formaldehyde/polyvinyl butyral matrix (70% PF/30% PVB) reinforced with Kevlar^®^ fibers through comprehensive in-plane tensile testing. Cyclic loading–unloading tests were conducted at a 100%/min strain rate using a universal testing system at room temperature on $\left(0_{4}\right)$, $\left(90_{4}\right)$, and ${\left(\pm 45\right)}_{s}$ laminates. The experimental results revealed significant nonlinear hardening behavior beyond yield stress, accompanied by yarn stiffening effects during loading cycles. A novel elastoplastic constitutive model was developed, incorporating Hill’s yield criterion adapted for orthotropic materials and an isotropic hardening function that accounts for equivalent plastic strains and progressive yarn stiffening. Laminates with other stacking sequences were also tested and the accuracy of the predictions of the nonlinear behavior was assessed. In these laminates, delaminations took place and the model provided an overestimation of the stress–strain response. Since the model could not predict delamination onset and propagation, an adaptation of the model considering fully delaminated interfaces brought a lower bound of this response. Despite the limitations of the model, it can be used to provide reasonable limits to the stress–strain response of laminates accounting for plastic strains within plies. This study provides essential mechanical properties and constitutive relationships for designing Kevlar^®^ composite structures with tailored stiffness characteristics for impact-resistant applications.

## 1. Introduction

Composite materials have revolutionized modern engineering through their exceptional strength-to-weight ratios and superior corrosion resistance compared to conventional metallic materials. Polymers reinforced with fibers, in particular, have seen remarkable growth and adoption across diverse industries, including aerospace, renewable energy, automotive, marine, sporting goods, and personal protection equipment [1,2]. Among these applications, Kevlar^®^-reinforced composites occupy a unique position due to their outstanding impact resistance and energy absorption capabilities, making them particularly valuable for ballistic protection and crashworthiness applications. The widespread adoption of these materials across diverse industries necessitates a comprehensive understanding of their mechanical behavior under various loading conditions, particularly in scenarios involving high strain rates and cyclic loading where nonlinear material responses become critical.

A comprehensive literature review reveals that existing studies on Kevlar^®^ composite laminates can be categorized into two primary geometric configurations: rectangular laminates [3,4,5,6,7,8] and military helmets [9,10,11,12], with research predominantly focused on ballistic impact analysis through numerical simulations (Table 1). However, these studies consistently treat Kevlar^®^ composites as brittle materials exhibiting purely elastic behavior until fracture employing linear elastic models that require nine compliance matrix constants: elastic moduli $\left(E_{L},\;E_{T},\;E_{N}\right),$ shear moduli $\left(G_{LT},\;G_{TN},\;G_{LN}\right),$ and Poisson’s ratios $\left(\nu_{TL},\;\nu_{NL},\;\nu_{NT}\right)$. Beyond these elastic constants, the characterization framework requires additional strength parameters encompassing both in-plane and through-thickness failure modes, necessitating a minimum of six strength constants to capture tensile, compressive, and shear failure mechanisms in each principal direction. This comprehensive material property database, comprising a minimum of 15 fundamental constants coupled with an appropriate failure criterion, theoretically enables the prediction of material failure under complex loading conditions.

The linear elastic assumption presents a fundamental limitation in modeling woven fabric behavior under projectile impact and represents a significant oversimplification of the actual material response. Experimental studies by Canceco et al. [13] demonstrate that materials exhibit predominantly nonlinear behavior beyond yield stress, contradicting this assumption; however, in this first study the quantification of elastic and plastic strains were not done. This nonlinear behavior is particularly pronounced in woven composites where complex interactions between matrix plastification, fiber reorientation, and progressive yarn straightening contribute to energy dissipation mechanisms. Understanding both elastic and plastic strain components is crucial because strain energy represents a key mechanism for dissipating the kinetic energy of projectiles in woven fabrics [14]. This misrepresentation of material behavior has critical implications for energy absorption calculations in impact-resistant design applications, potentially leading to unconservative designs that fail to adequately protect against ballistic threats or impact scenarios.

Current literature exhibits several critical deficiencies that limit the advancement of accurate Kevlar^®^ composite modeling, significantly hindering the development of reliable predictive capabilities for engineering applications. The following systematic gaps have been identified through extensive literature analysis:

- Material characterization gaps: A significant deficiency pervades the literature, where most studies either lack comprehensive material characterization or completely omit experimental validation. As Nunes et al. [5] observed, this absence of thorough material characterization forces researchers to rely on disparate literature references for model calibration rather than developing systematic experimental datasets tailored to specific material systems and manufacturing processes. Even when characterization data are provided, critical manufacturing parameters including thermoforming pressure, temperature, and processing duration are frequently unreported, severely hampering reproducibility and independent validation efforts. This systematic lack of documentation creates a cascading effect that undermines the reliability of subsequent modeling and design applications, making it nearly impossible to establish confidence bounds for numerical predictions.
- Material specification ambiguity: The specific type of Kevlar^®^ fiber and matrix system employed are often unidentified or inadequately described, hindering comparative analysis and model transferability across different material systems. This ambiguity extends to weave architecture specifications, fiber surface treatments, and matrix composition ratios, all of which significantly influence mechanical response and failure mechanisms.
- Geometric parameter justification: Specimen dimensions (width, length, and thickness) are typically presented without technical justification based on material microstructure or testing standards, potentially affecting the validity and reproducibility of experimental results.

The historical foundation of Kevlar^®^ composite modeling can be traced to the pioneering work of van Hoof [3], who conducted the earliest comprehensive numerical simulation of ballistic impact on Kevlar^®^-reinforced phenolic laminates. Van Hoof utilized the mechanical properties reported by Guoqi et al. [15] for Kevlar^®^ 29/polyester composites characterized through quasi-static and dynamic testing protocols. To achieve correlation between experimental and numerical results, van Hoof adjusted the in-plane shear and normal tensile strength parameters without fundamental justification. Despite the limitations and age of this work, the mechanical properties reported by van Hoof continue to be used in numerical simulation studies of ballistic impact on polymer matrix composites reinforced with Kevlar^®^, highlighting the persistent lack of comprehensive material databases in the field.

Alternative modeling approaches have explored viscoelastic characterization of Kevlar^®^ composites. Shim et al. [16] presented a viscoelastic model based on a two-springs, one-damper system; however, their study was limited to a single-ply analysis in an unspecified fiber direction, without characterization or modeling of off-axis $\left(45{}^{\circ}\right)$ behavior. This limitation is particularly significant because understanding orthotropic directional behavior remains crucial for developing laminates with customized stiffness properties tailored to specific applications. The absence of comprehensive directional characterization prevents the development of predictive models capable of accurately representing arbitrary laminate configurations encountered in practical applications.

**Table 1:** Mechanical properties of Kevlar^®^ in rectangular plates and military helmets subjected to ballistic impact. Modified from [13].

	Rectangular Plate						Military Helmet				
Mechanical Property	Kevlar 29/Phenolic	Kevlar 29/Epoxy	Kevlar 29/Epoxy	Kevlar 29/Epoxy	Kevlar	Kevlar	Kevlar 129	Kevlar	Kevlar	Kevlar 129	Kevlar 29
E_L_ [GPa]	18.5	18.5	7.618	10.06	18.5	18.5	22	18.5	18	22	18.5
E_T_ [GPa]	18.5	18.5	11.05	10.06	18.5	18.5	22	18.5	18	22	18.5
E_N_ [GPa]	1.47 ^(1)^ & 6.0 ^(2)^	6.0	6.0	6.0	6	6	9	6.0	4.5	9	6.0
ν_LT_	0.25	0.25	0.18	0.25	0.25	0.25	0.25	0.25	0.25	0.25	0.25
ν_LN_ = ν_NT_	0.33	0.33	0.33	0.33	0.33	0.33	0.33	0.33	0.33	0.33	0.33
G_LT_ [GPa]	0.77	0.77	2.123	0.77	5.43	5.43	0.77	0.77	0.77	0.77	0.77
G_LN_ [GPa]	2.715	5.43	5.43	5.43	5.43	5.43	5.34	2.715	2.6	2.715	2.72
G_TN_ [GPa]	2.715	5.43	5.43	5.43	0.77	0.77	5.34	2.715	2.6	2.715	2.72
X_LT_ [MPa]	555 ^(4)^	1850 ^(3)^	400	425			800	555	555	800	555
X_LC_ [MPa]	-	185	94	185			80	-	555	60	1200
X_TT_ [MPa]	555 ^(4)^	1850 ^(3)^	530	425			800	555	555	800	555
X_TC_ [MPa]	-	185	113	185			80	-	555	60	1200
X_NT_ [MPa]	1200	-	-	-			1200	1200	1050	-	1200
X_NC_ [MPa]	-	-	-	-			1200	-	1050	-	1200
S_LT_ [MPa]	77	-	-	-			77	77	77	77	77
S_LN_ [MPa]	1086	-	-	-			898	1086	1060	898	1086
S_TN_ [MPa]	1086	-	-	-			898	1086	1086	898	1086
S_n_ [MPa]	34.5	-	62.8	34.5				34.5	823.2 (4 plies)	34.5	-
S_s_ [MPa]	9	-	22.9	9				9.0	588 (4 plies)	-	-
S_c_ [MPa]	-	77	67	77				-	-	-	-
ρ [kg/m^3^]	1230	1440	1191	1025		1230	1230	1230	1230	1230	1230
Author	van Hoof [3]	Bresciani et al. [4]	Nunes et al. [5]	Scazzosi et al. [6]	Li et al. [7]	Umbharatwala and Goel [8]	Gower et al. [12]	Lee and Gong [11]	Tan et al. [9]	Li et al. [17]	Caçoilo et al. [10]
Concepts											
E_L_ = Young’s modulus in L direction E_T_ = Young’s modulus in T direction E_N_ = Young’s modulus in N direction ν_LT_ = Poisson’s ratio LT ν_LN_ = ν_NT_ = Poisson’s ratio LN & NT			G_LT_ = Shear modulus in LT direction G_LN_ = Shear modulus in LN direction G_TN_ = Shear modulus in TN direction X_LT_ = Tensile strength in the L direction X_TT_ = Tensile strength in the T direction			X_LC_ = Compression strength in the L direction X_TC_ = Compression strength in the T direction X_NT_ = Tensile strength in the N direction X_NC_ = Compression strength in the N direction S_LT_ = Shear strength in LT direction			S_LN_ = Shear strength in LN direction S_TN_ = Shear strength in TN direction S_n_ = Interface strength in N direction S_s_ = Shear strength in the interface S_c_ = Shear strength in LT direction ρ = Density		

^(1)^ Quasi-static tests; ^(2)^ dynamic tests conducted by Guoqi et al. [15] on a polyester matrix reinforced with Kevlar^®^ 29; ^(3)^ strength property “initially” adopted in the numerical model for the polymer matrix reinforced Kevlar^®^ by van Hoof [3]; ^(4)^ strength property “finally” adopted in the numerical model for the polymer matrix reinforced Kevlar^®^ by van Hoof et al. [18]; longitudinal (L); transversal (T); normal (N).

Investigations of stress–strain behavior under unloading–reloading cycles to determine irreversible strains in Kevlar^®^ composites are notably scarce in the literature, representing a critical knowledge gap for understanding energy dissipation mechanisms. Audibert et al. [19] characterized a woven Kevlar^®^/natural fibers/epoxy composite in 0°, 90°, and ${\left(\pm 45\right)}_{s}$ directions through cyclic tensile testing, controlling plastic flow using Hill’s yield criterion. However, their study did not present the hardening function describing the nonlinear behavior beyond yield stress; only the stiffness degradation during unloading cycles was presented. This incomplete characterization limits the applicability of their findings to predictive modeling applications where accurate representation of both hardening and degradation phenomena is essential.

In contrast, extensive research on nonlinear post-yield behavior exists for carbon and glass fiber composites, providing a foundation for analogous developments in Kevlar^®^ systems. Sun and Chen [20] analyzed in-plane nonlinear behavior using flow rule with Hill’s yield criterion for anisotropic materials, establishing fundamental approaches for orthotropic plasticity modeling. Goyal et al. [21] developed an elastoplastic model for polymer matrix reinforced with braided yarns from a microscopic perspective, demonstrating the feasibility of multi-scale modeling approaches. Vogler et al. [22] created an elastoplastic model for transversely isotropic materials under triaxial loading, coupled with a smeared crack model previously developed by Camacho et al. [23], showing the integration of plasticity and damage mechanics. Tan and Falzon [24] developed a model that describes the nonlinear behavior, stiffness degradation, and fracture process in PEEK thermoplastic matrix reinforced with AS4 carbon fibers under pure shear loading, observing shear modulus degradation through ASTM D7078/D7078M tests with unloading–reloading cycles. Paepegem et al. [25] demonstrated successful prediction of stress–strain curves for epoxy matrix reinforced with glass fibers in ${\left(\pm 45\right)}_{2s}$ and $\left(10_{8}\right)$ laminates, considering material plasticity and shear modulus degradation, although their model could not predict laminate rupture. These developments in related composite systems highlight both the potential and the challenges associated with developing comprehensive elastoplastic models for Kevlar^®^ composites.

Considering the information presented above, this investigation addresses the identified knowledge gaps by developing a comprehensive elastoplastic constitutive model for Kevlar^®^ composite laminates through systematic experimental characterization and theoretical development. The primary objectives include:

1. Fundamental characterization: Developing a robust experimental methodology to characterize in-plane behavior through controlled tensile tests with unloading–reloading cycles on symmetric laminates $\left(0_{4}\right)$, $\left(90_{4}\right)$, and ${\left(\pm 45\right)}_{s}$ directions. Virtually all the laminate volume is subjected to an in-plane stress state; through-thickness stresses are confined in the free-edge effect regions (these regions are less than 2 thicknesses wide [26]). For the in-plane modeling, characterizing the material behavior in different directions is essential due to the orthotropic nature of woven fabrics, which exhibit direction-dependent mechanical properties. This directional characterization will enable estimation of stress–strain response for any symmetric laminate configuration.
2. Plastic strain quantification: Systematically quantifying irreversible strains and material stiffening effects during cyclic loading to establish the foundation for energy absorption calculations in impact applications. This includes developing methodologies to separate elastic and plastic strain components and understanding the mechanisms responsible for progressive stiffness evolution.
3. Constitutive model development: Developing a constitutive model incorporating Hill’s yield criterion adapted for orthotropic materials and a hardening function accounting for equivalent plastic strains and progressive yarn stiffening effects. The model will capture both the nonlinear hardening behavior and the evolution of elastic properties observed experimentally.
4. Model validation and application: Validating the proposed model through comprehensive testing of various laminates with different fiber orientations, assessing prediction accuracy, and identifying limitations related to delamination and ultimate failure prediction. This validation will establish the model’s applicability range and provide guidance for practical implementation.

The significance of this study extends beyond academic understanding to practical engineering applications, particularly in the design of energy-absorbing structures and impact-resistant components where accurate prediction of plastic strain and energy dissipation is critical for performance optimization and safety assurance. The developed modeling framework will enable engineers to design Kevlar^®^ composite structures with tailored mechanical properties, optimize energy absorption characteristics for specific threat scenarios, and reduce the reliance on extensive experimental testing during the design process. Furthermore, the fundamental understanding of yarn stiffening mechanisms and plastic strain evolution will contribute to the broader knowledge base for textile composite behavior under extreme loading conditions.

## 2. Materials and Methods

### 2.1. Materials

#### 2.1.1. Fibers

**Figure 1:**
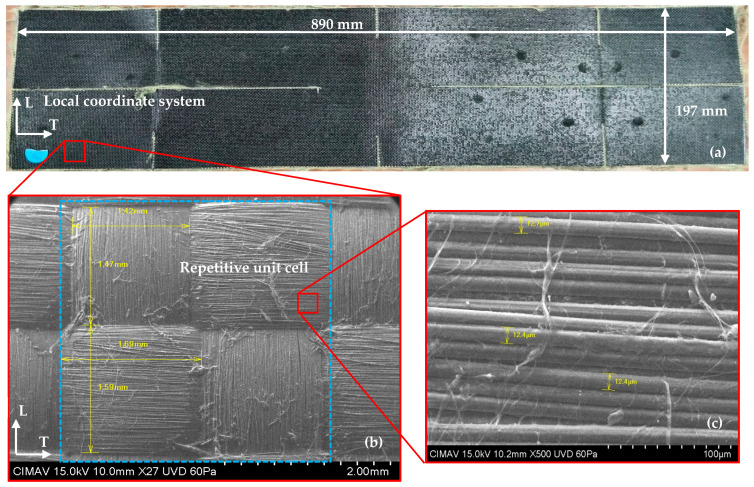
Material characterization of Kevlar^®^-29 reinforced composite: (**a**) as-received fabric layers with local coordinate system, (**b**) SEM image of repetitive unit structure, and (**c**) SEM image of individual fiber diameter measurement. Orthotropic directions of a woven fabric: *L* and *T* (modified from [13]).

The material under investigation consists of a two-dimensional woven Kevlar^®^-29 fabric (Dupont Corporation; Wilmington, DE, USA) characterized by interwoven warp and weft yarn systems (Figure 1a). Following standard composite material classification, this study deals with a polymer matrix composite reinforced with Kevlar^®^-29 fibers arranged in a plain weave pattern, where weft yarns alternately pass over and under warp yarns [27].

To ensure accurate material characterization, the orthotropic directions of the woven fabric were systematically identified through scanning electron microscopy (SEM) using a Hitachi^®^ SU3500 microscope (Hitachi Corporation; Tokyo, Japan). This analysis established a common 0° reference axis for all layers, with the warp direction designated as L direction and the weft direction as T. The microstructural analysis revealed a repetitive unit cell dimensions of 3 mm (Figure 1b), asymmetric yarn geometry with greater width in the T direction compared to the L direction, and a minimum individual fiber diameter of 12.4 μm (Figure 1c). It is worth mentioning that the material extraction was constrained by the presence of pre-existing horizontal and vertical cuts in the as-received fabric (Figure 1a), which limited specimen geometry options.

#### 2.1.2. Matrix

The Kevlar^®^ woven fabric employed a hybrid matrix system consisting of phenolic resin blended with polyvinyl butyral (PVB) in a composition ratio of 70% phenol formaldehyde (PF) and 30% PVB.

### 2.2. Methods

Following the established methodologies detailed in a previous study [13], the specimen characteristics and key methodological aspects are summarized below.

**Figure 2:**
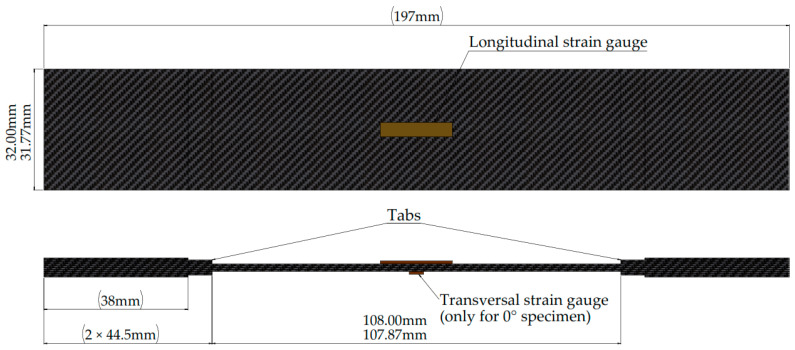
Geometry and thermoforming conditions of test specimens. Modified from [13].

Specimen design and preparation:

1. Geometry. Test specimens (Figure 2) were designed according to the ASTM D3039/D3039M standard [28], with dimensions optimized based on available raw material constraints (Figure 1a). Straight-sided specimen geometry was selected for all in-plane testing protocols.
2. Laminate. Three primary specimens were fabricated and tested: $\left(0_{4}\right)$, $\left(90_{4}\right)$, and ${\left(\pm 45\right)}_{s}$.
3. The methodology to determine the in-plane shear modulus $G_{LT}$, using a ${\left(\pm 45\right)}_{s}$ specimen was also presented in the study done by Canceco et al. [13].

Manufacturing process. Specimens were manufactured using a controlled thermoforming process with a Carver^®^ 4122 Manual Heated Press (Carver Corporation; Wabash, IN, USA) (10 t capacity) under the following conditions:

1. Primary consolidation: 192 °C, 10.12 MPa for 10.5 min.
2. Tab reinforcement: 192 °C, 20.67 MPa for 20 min (applied specifically to tab regions to prevent debonding during testing).

Instrumentation and testing setup:

1. Strain measurement. Specimens were instrumented with EP-08-500GB-120 strain gauges (Micro-Measurements^®^, Raleigh, NC, USA) for precise strain monitoring during testing.
2. Mechanical testing system. All tests were conducted using an Instron^®^ model 3382 universal testing system (Instron^®^, Norwood, MA, USA) equipped with mechanical wedge-action tension grips (type 2716-003) with a maximum load capacity of 10 t.

**Figure 3:**
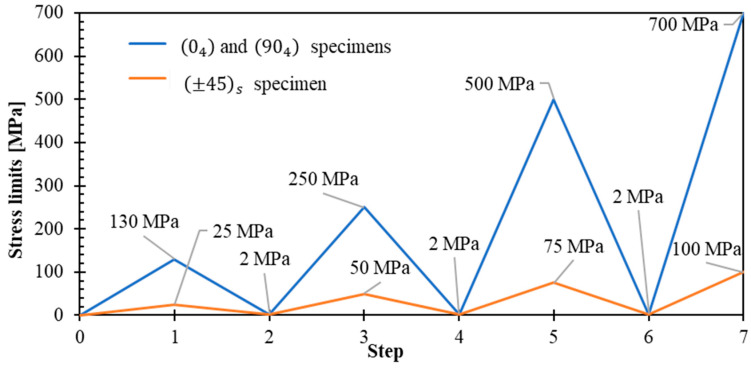
Stress-controlled unloading–reloading cycles at 100%/min for $\left(0_{4}\right)$, $\left(90_{4}\right)$, and ${\left(\pm 45\right)}_{s}$ specimens.

Testing protocol:

1. Loading conditions. All tensile tests employed strain-rate control with stress-delimited unloading–reloading cycles for $\left(0_{4}\right)$, $\left(90_{4}\right)$, and ${\left(\pm 45\right)}_{s}$ directions (Figure 3). Key testing parameters included:
  - Strain rate: 100%/min (selected to minimize viscous effects for ballistic application relevance while retaining a plasticity-based modeling approach). The universal testing system used is electromechanical equipment; therefore, higher strain rates cannot be achieved. Higher strain-rate testing would be beneficial and could be achieved using a split Hopkinson pressure bar (SHPB) or hydraulic devices (10^2^ s^−1^ to 10^4^ s^−1^ [29]); however, these equipment are not available in our laboratory. Dynamic testing on woven composites has been previously conducted using SHPB techniques [30,31], and the strain-rate dependent behavior of Kevlar^®^ fibers is well documented [32,33].
  - Minimum stress threshold: 2 MPa (established for safety to prevent compression-induced buckling).
  - Test environment: ambient laboratory conditions.
2. Cyclic loading strategy. The stress-controlled unloading–reloading protocol was specifically designed for each laminate orientation to characterize:
  - Elastic and plastic strain components.
  - Material stiffening effects during loading cycles.
  - Hysteresis behavior and energy dissipation mechanisms.

**Figure 4:**
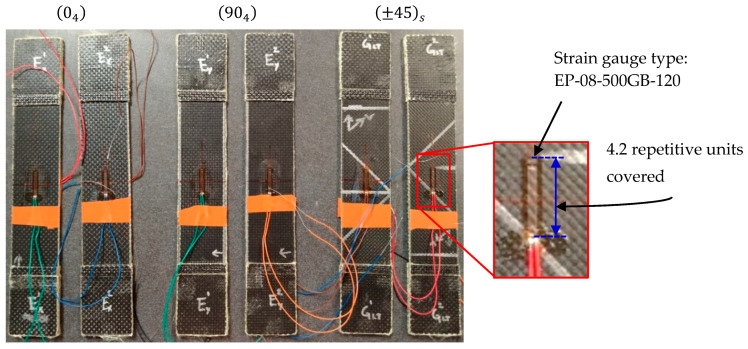
Instrumented specimens prepared for mechanical testing.

Figure 4 illustrates the final instrumented specimens prepared for mechanical testing, showing proper strain gauge placement. Two specimens were tested for each laminate orientation to assess result repeatability and material variability within the constraints of available material. While acknowledging that this sample size is insufficient for comprehensive statistical analysis, the consistent mechanical behavior trends observed across specimens provided adequate data for initial model development and validation. Future studies with larger sample sizes would be essential for establishing robust confidence intervals and comprehensive variability assessment.

This methodology provides a comprehensive framework for characterizing the elastoplastic behavior of Kevlar^®^ composite laminates under cyclic loading conditions, enabling the development of accurate constitutive models for engineering applications.

## 3. Results

**Figure 5:**
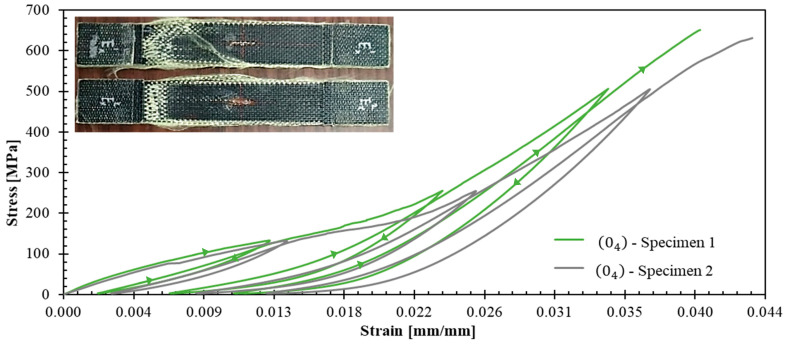
Stress–strain curves for $\left(0_{4}\right)$ specimens. Inset images illustrate the tested specimens.

**Figure 6:**
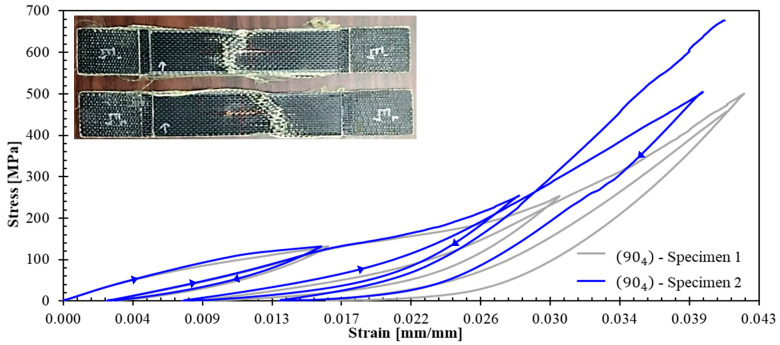
Stress–strain curves for $\left(90_{4}\right)$ specimens. Inset images illustrate the tested specimens.

The experimental campaign revealed distinct mechanical behaviors across different laminate configurations, providing detailed data for constitutive model development. Figure 5 and Figure 6 present the stress–strain curves with unloading–reloading cycles for $\left(0_{4}\right)$ and $\left(90_{4}\right)$ specimens, respectively. Strain measurements were obtained using strain gauges. The post-test appearance of the tested specimens is also documented in these figures.

Such curves (Figure 5 and Figure 6) demonstrated pronounced nonlinear hardening behavior following yield stress initiation, leading to plastic strain. This terminology aligns with established plasticity theory conventions, which in metallic materials is attributed to dislocation density increases [34]. However, for a polymer matrix composites reinforced with fibers, this nonlinear behavior can be attributed to a combination of matrix plastification, fiber reorientation mechanism, and progressive matrix-fiber debonding [35].

The observed dispersion in $\left(0_{4}\right)$ (Figure 5) and $\left(90_{4}\right)$ (Figure 6) specimens can be primarily attributed to the constrained specimen width, which accommodated only 10 repetitive unit cells. This geometric limitation introduces statistical variability that could be mitigated by increasing the specimen width to incorporate the maximum feasible number of repetitive units. However, specimen dimensions were constrained by available raw material geometry (Figure 1a). The maximum achievable specimen width compatible with the testing grip system is 50 mm, corresponding to approximately 16 repetitive unit cells.

**Figure 7:**
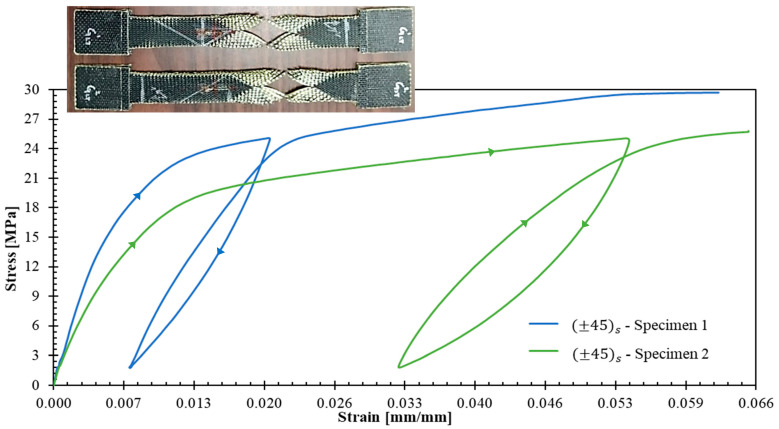
Stress–strain curves for ${\left(\pm 45\right)}_{s}$ specimens. Inset images illustrate the tested specimens.

Figure 7 presents the stress–strain response for ${\left(\pm 45\right)}_{s}$ specimens. The strain gauges failed prematurely due to excessive strains in the loading direction, coupled with significant transverse contraction that resulted in gauge debonding. Despite this instrumentation limitation, valuable initial data were obtained from the first unloading–reloading cycle, providing insight into the shear-dominated response of this laminate.

The dispersion observed in ${\left(\pm 45\right)}_{s}$ specimens (Figure 7) is considerably higher than that of $\left(0_{4}\right)$ and $\left(90_{4}\right)$ specimens; moreover, this increased dispersion is evident from the initial unloading–reloading cycle. This increased scatter could be reduced using the aforementioned specimen width increment strategy, as well as by implementing multi-plies such as ${\left({+45}_{2},{-45}_{2}\right)}_{s}$, which would yield improved laminate homogeneity and statistical consistency.

**Figure 8:**
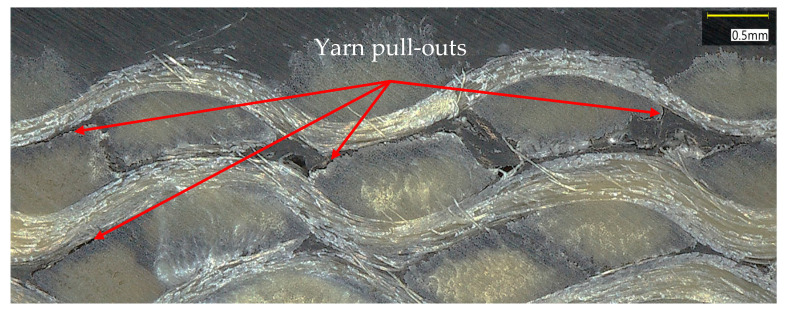
Micrography of a damaged specimen.

Hysteresis loops were consistently observed during each unloading–reloading cycle across all tested orientations: $\left(0_{4}\right)$, $\left(90_{4}\right)$, and ${\left(\pm 45\right)}_{s}$. This hysteretic response aligns with observations reported by Audibert et al. [19] and is primarily attributed to sliding friction phenomena at the matrix-fiber interphase, representing irreversible energy dissipation mechanism during cyclic loading. Figure 8 presents a micrography of a damaged specimen far from the ruptured region but close to the tab obtained with a VHX-7000 model Keyence^®^ digital microscope (Keyence Corporation, Osaka, Japan). As can be seen, there is clear evidence of yarn pull-out mechanisms that facilitate interfacial sliding and friction-based energy dissipation. This microstructural damage manifests as debonding at the yarn-matrix interface, enabling the relative displacement of individual yarns within the composite architecture and contributing to the observed hysteresis behavior during cyclic loading. In Appendix A, more images of a failed $\left(0_{4}\right)$ specimen obtained by X-ray tomography are shown.

Tensile testing consistently revealed intralaminar failure mechanisms across all three specimens, with no observable delamination. This failure mode indicates adequate interlaminar bonding strength relative to the in-plane strength properties for these specific laminates.

**Figure 9:**
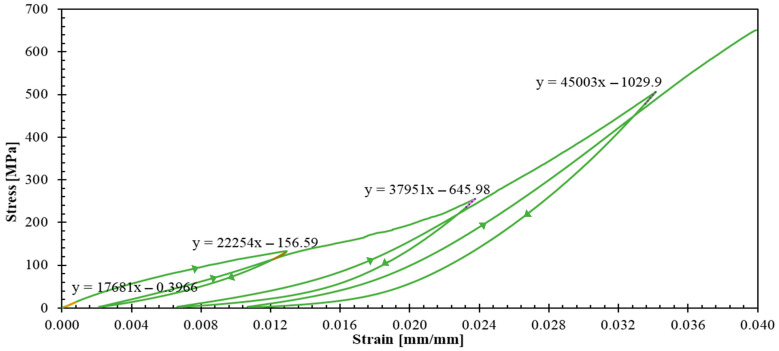
Stress–strain curve of a representative $\left(0_{4}\right)$ specimen.

**Figure 10:**
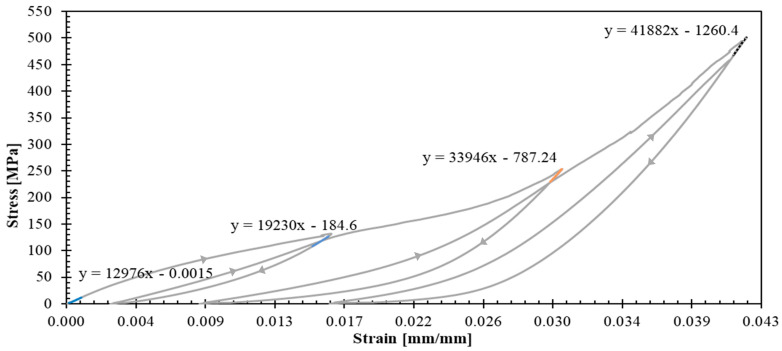
Stress–strain curve of a representative $\left(90_{4}\right)$ specimen.

**Figure 11:**
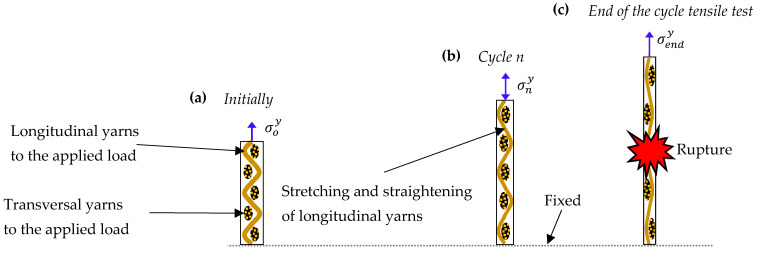
Progressive yarn straightening mechanism during cyclic tensile testing: (**a**) initial state with crimped longitudinal and transversal yarns, (**b**) intermediate straightening of longitudinal yarns under applied stress $\sigma_{n}^{y}$ during cycle n, and (**c**) final stage showing complete yarn alignment under applied stress $\sigma_{end}^{y}$ and leading to failure.

Representative curves for $\left(0_{4}\right)$ and $\left(90_{4}\right)$ specimens (Figure 9 and Figure 10) enabled quantification of unloading slopes at each cycle. These analyses revealed progressive slope increases during successive unloading events, indicating material stiffening behavior. This stiffening phenomenon provides evidence of yarn straightening and stretching mechanisms occurring throughout the tensile loading process until the ultimate failure. Figure 11 illustrates the gradual transition from yarn crimping to full extension through unloading–reloading cycles until ultimate tensile failure occurs.

The experimental data confirm that the 0° fiber direction corresponds to the warp direction of the woven fabric architecture, exhibiting superior stiffness characteristics. According to Ito and Chou [36], warp direction yarns experience greater initial tension during the weaving manufacturing process, while weft direction yarns exhibit undulation patterns above and below the warp yarns, resulting in reduced initial stiffness. This observation is consistent with Gower et al.’s research findings, which documented yarn straightening and stretching processes until ultimate failure [12].

**Figure 12:**
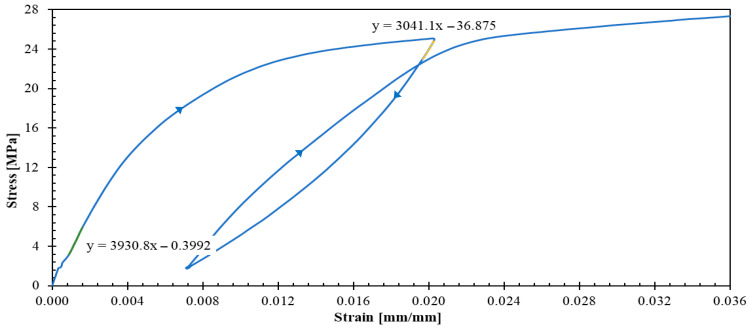
Stress–strain curve of a representative ${\left(\pm 45\right)}_{s}$ specimen.

Figure 12 reveals a contrasting mechanical response for the ${\left(\pm 45\right)}_{s}$ specimen, showing stiffness reduction during unloading phases. This behavior suggests laminate softening, fundamentally different from the stiffening observed in the fiber-dominated $\left(0_{4}\right)$ and $\left(90_{4}\right)$ specimens.

Evidence of progressive damage was inferred across all three specimens, manifested by the continuous sound emitted during the nonlinear behavior stage and the stress-whitening of the specimens. This behavior indicates accumulating microstructural damage affecting the material’s load-carrying capacity.

**Figure 13:**
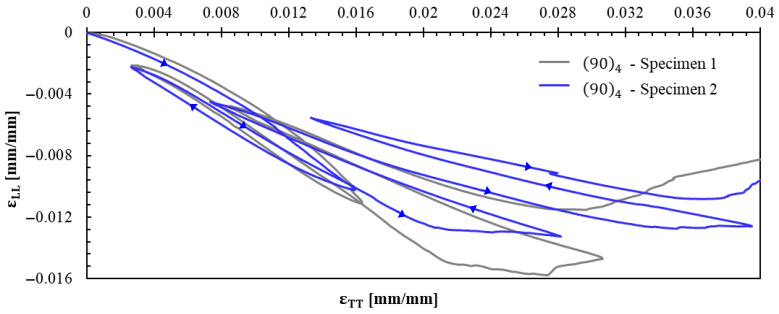
Axial strain–transversal strain with $\left(90_{4}\right)$ specimen.

Figure 13 shows the axial strain–transversal strain curves of specimens tested in tension using the $\left(90_{4}\right)$ specimens, from which an average Poisson’s ratio ($\nu_{TL}=0.399$) was calculated.

The comprehensive experimental characterization revealed an initial linear elastic behavior followed by predominantly nonlinear behavior beyond the yield stress threshold. The implementation of unloading–reloading cycles enabled clear differentiation between elastic and plastic strain components, providing quantitative data for a constitutive model calibration. Critical observations include material stiffening in the $0^{{}^{\circ}}$ and $90^{{}^{\circ}}$ principal directions due to progressive yarn straightening, contrasted with material stiffness reduction in the ${\left(\pm 45\right)}_{s}$ shear dominated configuration. Understanding plastic strain accumulation is fundamental for engineering applications, as these permanent deformations can compromise component functionality. Furthermore, comprehending the plastic response mechanisms is essential for energy absorption design in impact-resistant structures.

The experimental findings establish the foundation for developing a comprehensive elastoplastic constitutive model capable of representing the observed nonlinear behavior and progressive stiffness evolution in symmetric Kevlar^®^ composite laminates. This model framework will enable the design of laminates with customized stiffness characteristics tailored to specific application requirements, with relevance for impact-resistant structural components. The proposed model is presented and detailed below.

## 4. Elastoplastic Constitutive Model Development

Based on the experimental findings presented above, this section develops a simplified constitutive model that reproduces the main aspects of the experimentally observed mechanical behavior:

1. Strain hardening in specimens $\left(0_{4}\right)$ and $\left(90_{4}\right)$.
2. Nonlinear response beyond the yield stress with plastic strains in all the three tested laminates.
3. Progressive evolution of apparent elastic moduli—increasing in specimens $\left(0_{4}\right)$ and $\left(90_{4}\right)$ due to yarn straightening effects, while decreasing in specimen ${\left(\pm 45\right)}_{s}$.

The model framework deliberately excludes certain complex phenomena to maintain focus on the primary mechanical responses. Specifically, hysteresis effects during unloading–reloading cycles, progressive damage accumulation observed during each loading cycle, and delamination phenomena are not captured, as these require more sophisticated treatment involving damage mechanics and interface modeling that extends beyond the present study’s scope. The prioritization of yarn stiffening characterization over hysteresis reproduction reflects the model’s intended application in predicting primary load-carrying capacity and energy absorption potential rather than detailed cyclic response analysis.

**Figure 14:**
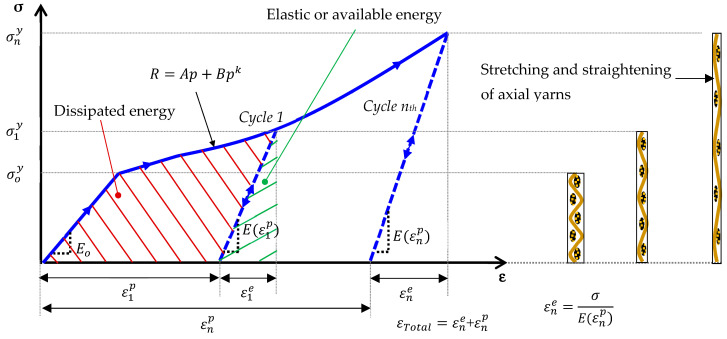
Proposed model with nonlinear hardening and stiffening for laminates $\left(0_{4}\right)$ and $\left(90_{4}\right)$.

Figure 14 illustrates the fundamental concepts of the proposed model with nonlinear hardening and stiffening for 0° and 90° laminates subjected to cyclic tensile tests. As shown in this figure, the stiffening is due to an alignment of fibers with the direction of the applied load; in the model, it is controlled by the axial plastic strain. For the $n_{th}$ cycle, $\varepsilon_{n}^{e}$ and $\varepsilon_{n}^{p}$ are the elastic strain and plastic strains, $\sigma_{n}^{y}$ is the apparent yield stress.

The constitutive formulation begins with the analysis of individual lamina behavior within the laminate structure. For each lamina, the following considerations were established:

1. Directions L and T represent the principal longitudinal and transversal orthotropy directions within the lamina plane, while direction N corresponds to the through-thickness direction. A plane stress state is adopted, which implies that $\sigma_{NN}=\sigma_{LN}=\sigma_{TN}=0$.
2. The Hill equivalent stress $\sigma^{eq}$ and initial yield stress $\sigma^{y0}$ are defined as follows [37].
  (1) $$\sigma^{eq}=\sqrt{\frac{3\left[F{\sigma_{TT}}^{2}+G{\sigma_{LL}}^{2}+H{\left(\sigma_{LL}-\sigma_{TT}\right)}^{2}+2N\sigma_{LT}^{2}\right]}{2\left(F+G+H\right)}}\;\;\;\;\;\;\;\;\;\;\;\;\;\;\;\;\;\;\;\;\;\;\;\;\;\;\;\;\;\;\;\;\;\;\;\;\;\sigma^{y0}=\sqrt{\frac{3}{2\left(F+G+H\right)}}$$
  where F, G, H, and N are parameters that depend on the yield stresses in the orthotropy directions and will be defined later.
3. A Hill plasticity criterion with hardening is adopted [37]. The yield function is:
  (2) $$F=\sigma^{eq}-R-\sigma^{y0}$$
  where R is the hardening function defined by:
  (3) $$R=Ap+Bp^{k}$$
  The hardening function presented in Equation (3) constitutes a first originality of the model, where, A, B, and k are hardening parameters that must be identified and p is the equivalent plastic strain and will be expressed later.
4. Associated plasticity is considered, which implies that the plastic potential is equal to the yield function [38]. A normal flow rule is assumed, so the infinitesimal variations of the plastic strain vector $\varepsilon^{p}={\left(\varepsilon_{LL}^{p},\varepsilon_{TT}^{p},\gamma_{LT}^{p}\right)}^{t}$:
  (4) $${\dot{\varepsilon }}^{p}=\dot{p}\;u$$
  where vector u is defined by:
  (5) $$u^{t}=\frac{3}{2\left(F+G+H\right)\sigma^{eq}}\left(G\sigma_{LL}+H\left(\sigma_{LL}-\sigma_{TT}\right)\;,\;F\sigma_{TT}+H\left(\sigma_{TT}-\sigma_{LL}\right)\;,\;2N\sigma_{LT}\right)\;$$
  where the superscript t denotes transposition.
5. The elastic behavior relates to the in-plane stress vector $\sigma ={\left(\sigma_{LL},\sigma_{TT},\sigma_{LT}\right)}^{t}$ with the elastic strain vector $\varepsilon^{e}={\left(\varepsilon_{LL}^{e},\varepsilon_{TT}^{e},\gamma_{LT}^{e}\right)}^{t}$:
  (6) $$\sigma =K\cdot \varepsilon^{e}$$
  where K is the elastic stiffness matrix in the material principal axes system, it is defined by:
  (7) $$K=\left(\begin{array}{ccc} \frac{E_{L}}{1-\nu_{LT}\nu_{TL}\;} & \frac{\nu_{LT}E_{T}}{1-\nu_{LT}\nu_{TL}} & 0\\ \frac{\nu_{LT}E_{T}}{1-\nu_{LT}\nu_{TL}} & \frac{E_{T}}{1-\nu_{LT}\nu_{TL}\;} & 0\\ 0 & 0 & G_{LT} \end{array}\right)$$
6. The plasticity-induced changes in elastic moduli and Poisson’s ratios ($\varepsilon_{LL}^{p}\geq 0$, $\varepsilon_{TT}^{p}\geq 0$) are modeled according to the following expressions (Equation (8)):
  (8) $$E_{L}=E_{L}^{0}+E_{L}^{1}{\left(\varepsilon_{LL}^{p}\right)}^{q}\;\;\;\;\;\;\;\;\;\;\;\;\;\;\;\;\;\;\;\;\;\;\;\;\;\;\;\;\;E_{T}=E_{T}^{0}+E_{T}^{1}{\left(\varepsilon_{TT}^{p}\right)}^{q}\;\;\;\;\;\;\;\;\;\;\;\;\;\;\;\;\;\;\;\;\;\;G_{LT}=G_{LT}^{0}+G_{LT}^{1}\left|\gamma_{LT}^{p}\right|\;\nu_{LT}=\nu_{LT}^{0}\left(1+\frac{E_{L}^{1}}{E_{L}^{0}}{\left(\varepsilon_{LL}^{p}\right)}^{q}\right)\;\;\;\;\;\;\;\;\;\;\;\;\;\;\;\;\;\;\;\;\;\;\;\;\;\;\;\;\nu_{TL}=\frac{\nu_{LT}^{0}E_{T}^{0}}{E_{L}^{0}}\left(1+\frac{E_{T}^{1}}{E_{T}^{0}}{\left(\varepsilon_{TT}^{p}\right)}^{q}\right)$$
  where $E_{L}^{0}$, $E_{T}^{0}$, $G_{LT}^{0}$, and $\nu_{LT}^{0}$ are elastic properties derived from the initial slope of the axial stress–strain curves from laminates $\left(0_{4}\right)$, $\left(90_{4}\right)$, and ${\left(\pm 45\right)}_{s}$; $E_{L}^{1}$, $E_{T}^{1}$, $G_{LT}^{1}$, and q are stiffening parameters that must be determined. It is worth mentioning that damage is not considered in the model and to reproduce the decrease in the apparent modulus of the ${\left(\pm 45\right)}_{s}$ laminate, $G_{LT}^{1}$ must be negative.
7. The total strain vector $\varepsilon ={\left(\varepsilon_{LL},\varepsilon_{TT},\gamma_{LT}\right)}^{t}$ is equal to the sum of the elastic $\varepsilon^{e}$ and plastic $\varepsilon^{p}$ strain vectors.
8. In an infinitesimal plastic process, the variation of equivalent plastic strain p is derived from the consistency condition $\dot{F}=0$, the flow rule (Equation (4) and Equation (5)), and the elastic behavior (Equation (6)):
  (9) $$\dot{p}=\frac{u^{t}\cdot \left(K\cdot {\dot{\varepsilon }}^{e}\right)}{\left(A+Bp^{k}-u^{t}\cdot \left[\left(\frac{\partial K}{\partial \varepsilon_{LL}^{p}}\cdot u_{1}+\frac{\partial K}{\partial \varepsilon_{TT}^{p}}\cdot u_{2}+\frac{\partial K}{\partial \gamma_{LT}^{p}}\cdot u_{3}\right)\cdot \left(K^{-1}\cdot \sigma \right)-K\cdot u\right]\right)}\;$$
  To identify the model parameters, first, it is useful to observe that, according to Figure 13, for tests with $\left(90_{4}\right)$, the plastic deformation variations ${\dot{\varepsilon }}_{LL}^{p}≃0$. According to Equation (8) and Equation (9), in those tests one obtains $\frac{{\dot{\varepsilon }}_{LL}^{p}}{{\dot{\varepsilon }}_{TT}^{p}}=-\frac{H}{F+H}$, which means that one should chose H≃0. The parameters F, G, and N that appear in the yield function (Equation (2)) are related to the initial yield stresses $\sigma_{LL}^{y0}$, $\sigma_{TT}^{y0}$, and $\sigma_{LT}^{y0}$ that would be directly measured in tensile tests with laminates $\left(0_{4}\right)$ and $\left(90_{4}\right)$ and with a shear test with a laminate $\left(0_{4}\right)$ (these latter tests were not performed in this work):
  (10) $$F=\frac{1}{{\left(\sigma_{TT}^{y0}\right)}^{2}}\;\;\;\;\;\;\;\;\;\;\;\;\;\;\;\;G=\frac{1}{{\left(\sigma_{LL}^{y0}\right)}^{2}}\;\;\;\;\;\;\;\;\;\;\;\;\;\;\;\;\;\;\;\;N=\frac{1}{2{\left(\sigma_{LT}^{y0}\right)}^{2}}$$
  The computational implementation employs classical laminated plate theory for symmetric laminates under uniaxial tension, with the complete system of equations solved using SCILAB software. Parameter identification follows a least-squares optimization approach, fitting theoretical predictions to experimental data from representative stress–strain curves (Figure 9, Figure 10 and Figure 12) and apparent elastic moduli measured during unloading cycles. The parameters A, B, k, $\sigma_{LL}^{y0}$, $\sigma_{TT}^{y0}$, $\sigma_{LT}^{y0}$, $E_{L}^{1}$, $E_{T}^{1}$, $G_{LT}^{1}$, and q are identified and presented in Table 2.

**Figure 15:**
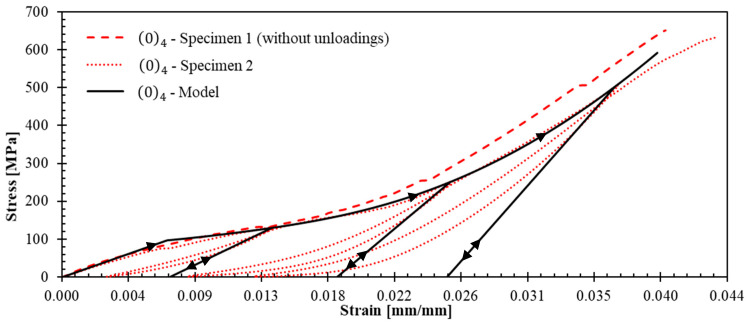
Stress–axial strain curves for $\left(0_{4}\right)$ laminates: experiments and theoretical prediction.

**Figure 16:**
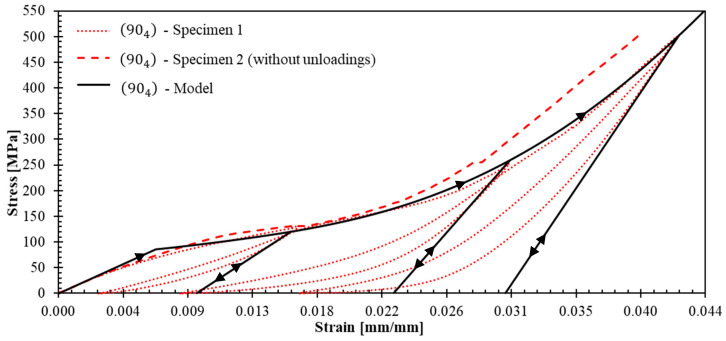
Stress–axial strain curves for $\left(90_{4}\right)$ laminates: experiments and theoretical prediction.

**Figure 17:**
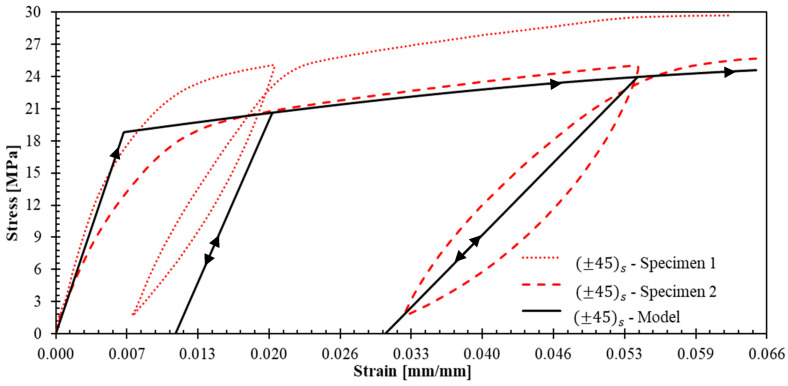
Stress–axial strain curves for ${\left(\pm 45\right)}_{s}$ laminates: experiments and theoretical prediction.

Figure 15, Figure 16 and Figure 17 show the theoretical stress–strain curves obtained for laminates $\left(0_{4}\right)$, $\left(90_{4}\right)$, and ${\left(\pm 45\right)}_{s}$. The experimental results are also presented in these figures; the unloading–reloading paths are only displayed for the tests that were used to fit the theoretical results (specimens 2, 1 and 2 for laminates $\left(0_{4}\right)$, $\left(90_{4}\right)$, and ${\left(\pm 45\right)}_{s}$, respectively). The model demonstrates good agreement with the behavior of the three laminates: R^2^ values for $\left(0_{4}\right)$, $\left(90_{4}\right),$ and ${\left(\pm 45\right)}_{s}$ are 0.951, 0.998, and 0.926, respectively. The R^2^ values were obtained from the representative curves without unloading because it is not the purpose of the model to reproduce the experimental nonlinearity observed in the unloading paths and the hysteresis behavior, as it does not account for friction, and damage. Importantly, the model successfully replicates the stiffness increase and strain hardening observed in tests $\left(0_{4}\right)$ and $\left(90_{4}\right)$, as well as the elastic modulus reduction in test ${\left(\pm 45\right)}_{s}$.

## 5. Model Accuracy Assessment for Alternative Laminates

Since the model parameters were obtained by fitting the response of $\left(0_{4}\right)$, $\left(90_{4}\right)$, and ${\left(\pm 45\right)}_{s}$ laminates, it is necessary to try the model accuracy in predicting the behavior of other laminates subjected to tensile loads. The 6-ply and 8-ply laminates were selected as ${\left(90,\pm 45\right)}_{s}$ and ${\left(\pm 70,\pm 35\right)}_{s}$, respectively.

**Figure 18:**
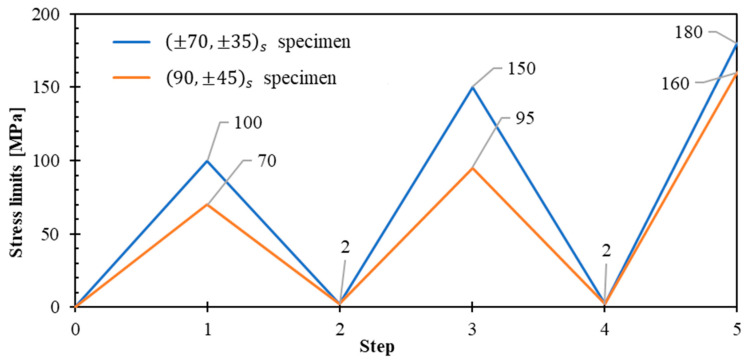
Stress-controlled unloading–reloading cycles at 100%/min for ${\left(\pm 70,\pm 35\right)}_{s}$ and ${\left(90,\pm 45\right)}_{s}$ specimens.

Figure 18 presents the stress limits for the alternative specimens. The predictions were determined using the model. Both laminates were designed as symmetric laminates (B = 0) to eliminate bending-extension coupling effects, preventing the generation of moments at the specimen grip interfaces during tensile testing and avoiding potential specimen twisting.

**Figure 19:**
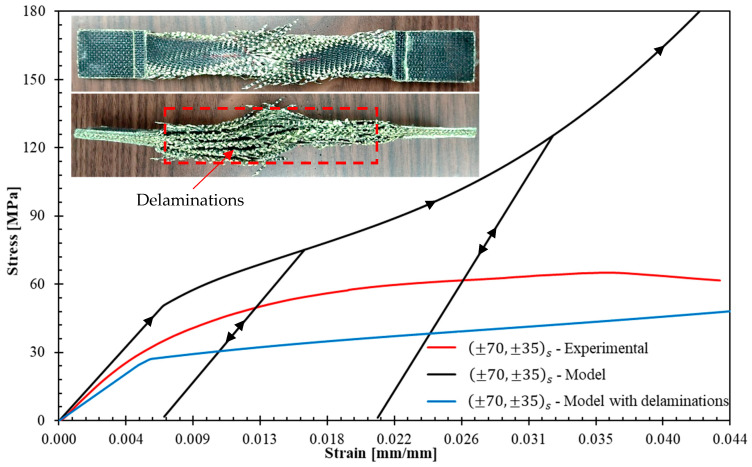
Comparison between model predictions and experimental results for ${\left(\pm 70,\pm 35\right)}_{s}$ laminate. The upper images show the experimentally observed delaminations.

**Figure 20:**
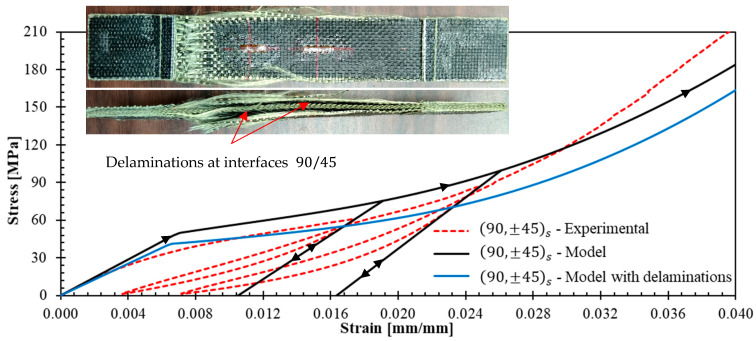
Comparison between model predictions and experimental results for ${\left(90,\pm 45\right)}_{s}$ laminate. Inset images illustrate the symmetric delamination pattern observed experimentally.

Figure 19 and Figure 20 presents the stress–strain curves obtained from ${\left(\pm 70,\pm 35\right)}_{s}$ and ${\left(90,\pm 45\right)}_{s}$ experiments alongside those predicted by the model (black curves). The model correctly predicts the initial stiffness in the two cases. However, the quality of the predictions in all the load history is not as good as for $\left(0_{4}\right)$, $\left(90_{4}\right)$, and ${\left(\pm 45\right)}_{s}$ laminates. This is attributed to delaminations occurring in the ${\left(\pm 70,\pm 35\right)}_{s}$ and ${\left(90,\pm 45\right)}_{s}$ laminates. The model does not consider delamination onset and propagation, and for a given strain, the stress predictions should overestimate the measured ones. This overestimation is verified in both specimens, except for axial strains greater than 0.03 in the ${\left(90,\pm 45\right)}_{s}$ laminate. This exception can be explained by experimental scattering; for $\left(90_{4}\right)$ laminates, the model also underestimated the stresses of specimen 2 for strains greater than 0.02 (see Figure 16).

Let us provide a brief description of the delamination process observed in the experiments. In laminate ${\left(\pm 70,\pm \;35\right)}_{s}$, delamination was visually observed at the free edges shortly after test initiation and propagated rapidly toward the laminate center. Delamination at the +70/−70 and +35/−35 interfaces was evident. In laminate ${\left(90,\;\pm 45\right)}_{s}$, delamination occurred at the 90/+45 interfaces and propagated slowly toward the center. To confirm that high stresses develop at the interfaces mentioned above, the DEILAM software [26] was employed to calculate edge effects on interlaminar stresses. This software employs a layerwise model [39] that assumes linear layer behavior and has been used to predict the onset of delamination in laminates without significant plastic strains within layers [40]. The application of DEILAM to the material studied here is limited to identifying the laminate interfaces most susceptible to delamination. Both specimens were subjected to an axial strain level of ε=0.1%. The through-thickness elastic properties required in this software to perform the calculations are $E_{N}=0.75\;GPa$, $G_{LN}=G_{TN}=0.0913\;GPa$, $\nu_{LN}=1.22$, and $\nu_{TN}=1.04$; these properties were obtained from [41]. Table 3 shows the maximum interface stresses in the laminates obtained with DEILAM. It should be noted that interface stresses are zero for laminates $\left(0_{4}\right)$ and $\left(90_{4}\right)$ and less than 0.01 MPa for ${\left(\pm 45\right)}_{s}.$ The calculations demonstrate that the +70/−70 interfaces in laminate ${\left(\pm 70,\pm \;35\right)}_{s}$ are most prone to delamination, followed by the +35/−35 interface and the 90/45 interface in laminate ${\left(90,\;\pm 45\right)}_{s}$. This is consistent with experimental observations.

At this stage, for laminates that can exhibit delaminations, the model can only be used to provide an upper bound to the stress for a given axial strain. Let us now propose an adaptation of the model to bring a lower bound. For a laminate that exhibits progressive delamination at critical interfaces, the model can be used assuming that these critical interfaces are completely damaged. The laminate is modeled by a set of decoupled sub-laminates working in parallel. If one applies this technique to the tested laminates:

1. Laminate ${\left(\pm 70,\pm 35\right)}_{s}$ with delaminations is modeled by the set of the following five parallel sub-laminates: $\left(+70\right)$, $\left(-70,+35\right)$, $\left({-35}_{2}\right)$, $\left(+35,-70\right)$, and $\left(+70\right)$.
2. Laminate ${\left(90,\pm 45\right)}_{s}$ with delaminations is simulated by the set of three parallel sub-laminates: (90), ${\left(\pm 45\right)}_{s}$, and (90).

The response of each sub-laminate was simulated using the plasticity model. In Figure 19 and Figure 20, the results of this new modeling approach are added with the legend “Model with delaminations” (blue curves). The results confirm that in all cases, once the laminate enters the nonlinear behavior, the model with delaminations provides a lower bound for the experimental response.

Despite the presence of delaminations, the model and its adaptation can help to provide reasonable upper and lower bounds to the response of a laminate.

## 6. Conclusions

This study presents a comprehensive elastoplastic constitutive model for Kevlar^®^-reinforced composite laminates based on systematic experimental characterization and theoretical development. The developed model can be a valuable tool for designing Kevlar^®^ composite structures with tailored stiffness characteristics for impact-resistant applications, addressing critical gaps in current modeling approaches that treat these materials as purely brittle elastic. The following key findings and contributions emerge from this research:

1. Cyclic tensile tests at 100%/min strain rate revealed distinct mechanical behaviors: fiber-dominated directions ($0^{{}^{\circ}}$ and $90^{{}^{\circ}}$) exhibited nonlinear hardening and progressive yarn stiffening, whilst the shear-dominated specimen ${\left(\pm 45\right)}_{s}$ demonstrated a compliance increase. Hysteresis loops observed across all orientations indicate energy dissipation caused by damage of matrix/yarn interfaces and friction during the sliding of yarns within the matrix.
2. A novel elastoplastic model was successfully developed incorporating Hill’s yield criterion for orthotropic materials, a hardening function accounting for accumulated plastic strains, and progressive stiffening mechanisms, representing yarn straightening effects. The model accurately predicts stress–strain behavior for symmetric laminates with various ply orientations.
3. Validation tests with other laminates [${\left(\pm 70,\pm 35\right)}_{s}$ and ${\left(90,\pm 45\right)}_{s}$] showed good agreement between model predictions and experimental behavior. However, limitations include premature free-edge delamination in diverse orientations due to high interlaminar stresses, inability to predict ultimate failure. Despite these limitations, experimental curves remained within model predictions.
4. Future work will focus on determining interlaminar stresses using a layerwise theory, incorporating the through-thickness mechanical properties presented by Canceco et al. [41] and the findings from this manuscript. Delamination initiation and propagation can be simulated using a cohesive zone model, which requires additional parameters and more in-depth investigation. This comprehensive modeling strategy can be implemented in future studies using commercial finite element software.
5. Additional specimens are required for testing in all three directions to develop a statistically robust model with appropriate confidence intervals and variability assessment. Unfortunately, insufficient material is available to conduct these additional tests due to constraints in the as-received fabric geometry. Nevertheless, the model successfully captures key phenomena occurring in the material, including yarn stiffening during each unloading cycle and nonlinear behavior beyond the yield stress.

This research addresses critical gaps in Kevlar^®^ composite modeling by providing accurate elastoplastic characterization beyond linear-elastic assumptions, enabling energy absorption predictions for impact-resistant applications, and establishing fundamental mechanical properties for engineering design. Future research should focus on developing a delamination prediction and ultimate strength assessment model.

## Figures and Tables

**Table 2:** Elastoplastic properties.

$E_{L}^{0}$ [MPa]: 14 × 10^3^	$E_{L}^{1}$ [GPa]: 5.32 × 10^3^	$\sigma_{LL}^{y0}$ [MPa]: 97.7	A [GPa]: 2.88
$E_{T}^{0}$ [MPa]: 13 × 10^3^	$E_{T}^{1}$ [GPa]: 3.92 × 10^3^	$\sigma_{TT}^{y0}$ [MPa]: 85.8	B [GPa]: 3.41 × 10^5^
$G_{LT}^{0}$ [MPa]: 0.802 × 10^3^	$G_{LT}^{1}$ [GPa]: −9.0	$\sigma_{LT}^{y0}$ [MPa]: 9.5	q [-]: 1.4
$\nu_{LT}^{0}$ [-]: 0.36	–	–	k [-]: 4.1

**Table 3:** Interlaminar shear (τ) and normal ($\sigma^{n})$ stresses obtained by DEILAM assuming a linear elastic behavior and a 0.1% tensile strain.

-	Laminate						
Interface Stresses	${\left(\pm 70,\pm 35\right)}_{s}$				${\left(90,\pm 45\right)}_{s}$		
	+70/−70	−70/+35	+35/−35	−35/−35	90/+45	+45/−45	−45/−45
τ (MPa)	1.42	0.35	0.86	0.00	0.49	0.07	0.00
$\sigma^{n}$ (MPa)	0.30	0.01	0.02	0.02	0.02	0.04	0.04

**Figure A1:**
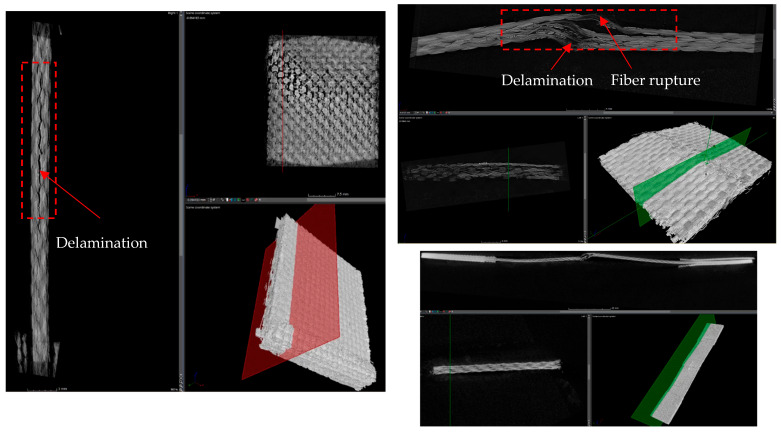
X-ray tomographic analysis of tested specimens revealing internal damage mechanisms. Images show delamination and fiber rupture patterns in different viewing orientations and 3D reconstructions.

## Data Availability

Data are contained within the article.

## Appendix A

X-ray computed tomography (CT) was performed using a Nikon^®^ XT-H-225 system (Nikon Corporation; Tokyo, Japan) on a tested specimen. To enhance visualization of failure mechanisms, tomographic scans were acquired in the grip-tab region and the central gauge sections of the specimen. The CT analysis revealed distinct damage modes including delamination and fiber rupture, as illustrated in the cross-sectional views and 3D reconstructions (Figure A1).
